# Not Just Another Scaffolding Protein Family: The Multifaceted MPPs

**DOI:** 10.3390/molecules25214954

**Published:** 2020-10-26

**Authors:** Agnieszka Chytła, Weronika Gajdzik-Nowak, Paulina Olszewska, Agnieszka Biernatowska, Aleksander F. Sikorski, Aleksander Czogalla

**Affiliations:** 1 Department of Cytobiochemistry, Faculty of Biotechnology, University of Wroclaw, 50-383 Wroclaw, Poland; agnieszka.chytla@uwr.edu.pl (A.C.); weronika.gajdzik@uwr.edu.pl (W.G.-N.); paulina.skibinska@uwr.edu.pl (P.O.); agnieszka.biernatowska@uwr.edu.pl (A.B.); 2 Research and Development Center, Regional Specialist Hospital, Kamieńskiego 73a, 51-154 Wroclaw, Poland; aleksander.sikorski@wssk.wroc.pl

**Keywords:** membrane palmitoylated protein (MPP), membrane-associated guanylate kinases (MAGUKs), membrane rafts, cell adhesion, cell polarity

## Abstract

Membrane palmitoylated proteins (MPPs) are a subfamily of a larger group of multidomain proteins, namely, membrane-associated guanylate kinases (MAGUKs). The ubiquitous expression and multidomain structure of MPPs provide the ability to form diverse protein complexes at the cell membranes, which are involved in a wide range of cellular processes, including establishing the proper cell structure, polarity and cell adhesion. The formation of MPP-dependent complexes in various cell types seems to be based on similar principles, but involves members of different protein groups, such as 4.1-ezrin-radixin-moesin (FERM) domain-containing proteins, polarity proteins or other MAGUKs, showing their multifaceted nature. In this review, we discuss the function of the MPP family in the formation of multiple protein complexes. Notably, we depict their significant role for cell physiology, as the loss of interactions between proteins involved in the complex has a variety of negative consequences. Moreover, based on recent studies concerning the mechanism of membrane raft formation, we shed new light on a possible role played by MPPs in lateral membrane organization.

## 1. Introduction

Membrane-associated guanylate kinases (MAGUKs) represent a diverse group of multidomain scaffolding proteins that are mainly present at sites of cell–cell contact and serve as platforms for numerous cellular processes including cell adhesion, cell signaling and cell polarity [1,2,3]. Through the years, the MAGUK family has expanded and currently consists of several subfamilies, one of which comprises membrane palmitoylated proteins (MPPs), which are the subject of this review.

**Figure 1:**
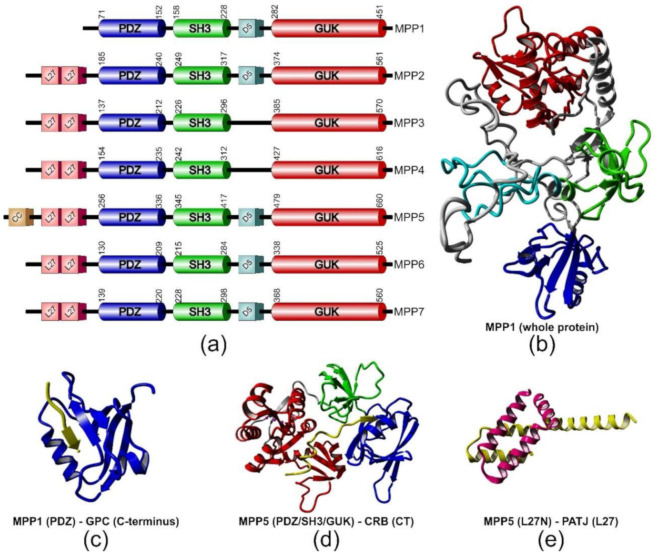
Structural features of membrane palmitoylated proteins (MPPs). (**a**) Schematic representation of domain structure of MPPs. The boundaries of the three canonical domains (cylinders) were estimated according to the UniProt database (www.uniprot.org). (**b**) Model of three-dimensional structure of MPP1 built with the I-TASSER web server [43]. Color code corresponds to the domains depicted in (**a**). Examples of complexes formed by MPPs with their partner proteins/protein fragments (in yellow); (**c**) the PDZ domain of MPP1 in complex with glycophorin C-terminal peptide (PDB ID: 2EJY) [44]; (**d**) the MPP5 PDZ/SH3/GUK tandem (for explanation of these abbreviations see the main text) bound to CRB–cytoplasmic tail (CT) (PDB ID: 4WSI) [25]; (**e**) the MPP5 L27N and Pals1-associated tight junction protein (PATJ) L27 heterodimer complex (PDB ID: 1VF6) [45].

MPPs are ubiquitously expressed, membrane-associated, palmitoylated proteins found at the cytosolic leaflet of the plasma membrane [4,5,6,7,8,9]. *S*-palmitoylation, the post-translational, reversible, covalent attachment of a palmitoyl acyl chain to a cysteine residue, is a characteristic feature of the MPP subfamily, which facilitates their membrane binding and may target proteins to membrane rafts [4,10]. MPPs were first discovered in *Drosophila* as the Stardust (Sdt) protein. Then, its homologs were found in vertebrates and renamed as MPPs. As of today, this group is represented by seven members, MPP1–MPP7 [11]. The multidomain structure is at the root of the diversity of functions played by MPPs, mainly through sequestration of specific molecules to particular regions of membranes. Each member of the family contains a highly conserved core composed of three sequentially arranged domains: a post synaptic density protein 95 (PSD-95)/disc large (Dlg)/zonula occludens-1 (ZO-1) (PDZ) domain, an Src homology 3 (SH3) domain and a catalytically inactive guanylate kinase (GUK) domain (Figure 1a) [1,12,13]. The PDZ domain has a major role in assembling protein complexes [14,15]. MPPs have only one PDZ domain with a length of approximately 80 amino acid residues (Figure 1a,b; depicted in dark blue) [16]. The most common type of PDZ ligand motif is the type II PDZ-binding motif, where a PDZ domain recognizes short amino acid sequences at the C-terminus of a ligand protein (Table 1). According to some reports, the PDZ domains may present the ability to interact with lipids and/or other proteins [17,18].

Another core domain of MPPs is the SH3 domain, which can be found in many proteins as a protein–protein interaction site [19,20,21,22]. The SH3 domain has the ability to form intramolecular and intermolecular interactions with the GUK domain [1,23,24,25,26], and this mechanism does not depend on SH3-mediated recognition of proline-rich sites [8]. Moreover, SH3-GUK interactions were demonstrated to serve as interaction modules for other MPPs and MAGUKs (Table 1).

Despite the high degree of sequence similarity to actual guanylate kinases, the GUK domain of MPPs shows no enzymatic activity. However, the binding site for ATP and GMP in MPPs (MPP1) is intact, which may suggest that nucleotides may have some regulatory role in GUK functions [12,27,28]. These assumptions seem to be confirmed by reports about bacterially expressed GUK domain of MPP1, which showed little catalytic activity [1,11]. Additionally, all MPPs, except MPP1, have two N-terminal Lin-7 binding domains, each with a length of approximately 55 amino acid residues (Figure 1a, depicted in pink), named L27 (Lin-2- and Lin-7-binding or Veli-binding domain), which were originally identified in *Caenorhabditis elegans* Lin-2 and Lin-7 proteins [29]. The L27 domain is capable of organizing multiple protein complexes through its ability to form heterotetramers. Most of them play essential roles in cell polarity, cell division or clustering of receptors [30,31,32,33]. The other characteristic domain that can be distinguished from the structure of several MPP subfamily members is D5, also known as HOOK, with a length of approximately 40 amino acid residues. (Figure 1a, depicted in light blue), via which MPPs bind to proteins from the 4.1-ezrin-radixin-moesin (FERM) protein family (Table 1).

Multidomain architecture allows MPPs to form various protein–protein complexes at the membrane–cytosol interface, thus making them important scaffolding molecules facilitating the assembly of multiprotein complexes involved in numerous cellular processes such as signal transduction, secretion, absorption, maintaining cell polarity or cell adhesion, which might be mediated by several types of cell junctions [34,35,36,37,38]. Notably, loss of control of any of these features may drive malignant transformations and might lead to invasion or metastasis [39,40,41,42].

In this review, we would like to collect available information about the MPP subfamily, considering their role in the formation of selected multiprotein complexes in the membranes. We highlight the universal character and specific features of MPP-mediated complexes in different cells and their impact on plasma membrane architecture/remodeling and cell physiology. We also discuss the recent findings of how MPPs contribute to the lateral plasma membrane organization by interacting with the raft proteins, flotillins.

## 2. MPPs in Complexes with FERM Family Members

Almost all proteins from the MPP family were proved or at least suggested to form complexes with FERM family members. This family includes over 40 proteins with a characteristic N-terminal FERM domain that serves as a binding site for a set of various membrane proteins and lipids, including MPPs (Table 1) [46]. The MPP-FERM complexes are often associated with transmembrane proteins (TMPs) that bind to MPPs via a type II PDZ-binding motif (Table 1). Generally, such MPP-FERM-TMP complexes function as bridges, linking the actin cytoskeleton to the plasma membrane of various cells.

**Figure 2:**
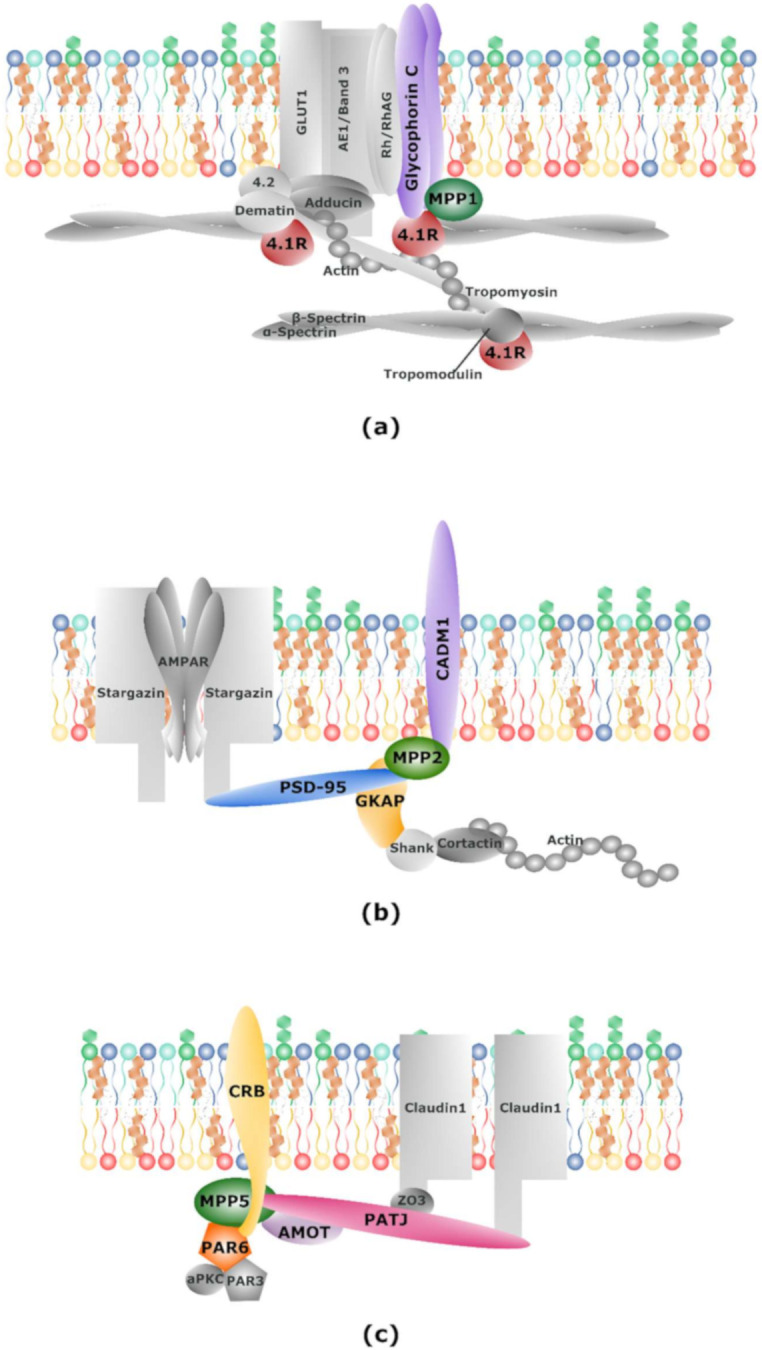
MPPs in protein complexes. Schematic illustration of protein complexes formed by MPPs. (**a**) Scaffolding ternary complex formed by MPP1, glycophorin C (GPC) and protein 4.1 found in erythrocyte membranes; (**b**) scaffolding complex formed by MPP2, postsynaptic density protein 95 (PSD-95), guanylate kinase-associated protein (GKAP) and CADM1 found at neuronal postsynaptic membrane; (**c**) Crumbs polarity complex formed by MPP5, CRB and PATJ and partitioning defective (Par) complex formed by Par6, Par3 and atypical protein kinase C (aPKC) found in epithelial cells. See main text for details.

One of the best-characterized protein families from the FERM domain group, in terms of interactions with MPPs, is the 4.1 proteins. There are four paralogs of 4.1 protein identified in vertebrates, which are named after the initial studies of tissue/organ localization—4.1R (“restricted”, red cell), 4.1B (brain), 4.1G (general) and 4.1N (neuronal) [93]. Similar to MPPs, 4.1 proteins are ubiquitously expressed and due to their domain structure, they serve as adaptors for a set of TMP and cytoskeletal proteins [46,93]. The first discovered member of the 4.1 family, protein 4.1R, forms in erythrocytes a ternary complex together with a member of the MPP family, MPP1, and a transmembrane glycoprotein named glycophorin C (GPC) (Figure 2a) [16,47,48,49,50,51,52]. The FERM domain of protein 4.1R was mapped as an interaction site of the D5 domain of MPP1, which is characterized by a cluster of lysine residues (KKKKYKDK) (Table 1) [48,50,51,58]. Further investigation reported two distinct binding sites and the D5 domain was shown to bind a 31-amino acid residue sequence, encoded by exon 10, and to a 35-residue region, encoded by exon 5, both located in the FERM domain of protein 4.1 [47,58]. Moreover, the second binding site was of higher affinity and the presence of a sequence stretch originated from the alternatively spliced exon 5 was crucial for plasma membrane localization of protein 4.1R in epithelial cells [58]. The dissociation constants estimated by Hemming et al. implied that interaction through MPP1 is of higher affinity than binding between GPC and protein 4.1R alone [50]. However, further studies showed that 4.1R increases the strength of interaction between MPP1 and GPC [47]. Notably, the binding of calmodulin to protein 4.1R decreased the affinity of this protein toward other proteins of the ternary complex in a Ca^2+^-dependent manner. In the same study, MPP1 and GPC were shown to have no impact on the mutual interaction between 4.1R-GPC and MPP1-4.1R, respectively [47]. In the case of GPC, the MPP1 interaction site was mapped to the N-terminal 21 amino acid residues of its PDZ domain [49]. This part was shown to bind the E-Y-F-I motif found in the cytoplasmic domain of GPC, where I128 (P0) and Y126 (P2) were indicated to be crucial for this interaction (Figure 1c). Moreover, NMR studies suggested that the interaction between the MPP1 PDZ domain and the C-terminal motif of GPC resembles the ligand-binding mode of other PDZ domains [16]. Quite possibly, this binding involves interactions between the residues in the MPP1’s hydrophobic pocket and GPC I128, and MPP1’s V130 and/or Q134 and GPC Y126 [44].

The MPP1-4.1R-GPC ternary complex anchors the spectrin-actin skeleton to the erythrocyte membrane bilayer and thus functions in maintaining the extraordinary elasticity and mechanical stability of the erythrocyte (Figure 2a, for review see [94]). The loss of MPP1 protein was detected in the case of hereditary and Leach elliptocytoses, which were characterized by the lack of protein 4.1R and GPC, respectively [95]. Generally, in both disorders, the disturbance of the MPP1-4.1R-GPC complex affected the mechanical properties of the erythrocyte membrane skeleton, resulting in the elliptical shape of red blood cells (RBCs) [95,96]. Recently, a study was presented where partial deletion of the *MPP1* gene was the probable cause of developmental defects in a pediatric patient who suffered from severe hemophilia A [97]. Western blot analysis of MPP1 showed a complete lack of its expression, which seemed to have no impact on other proteins of the erythrocyte membrane skeleton, including protein 4.1R. However, this conclusion was based only on the result of Coomassie staining of electrophoretically separated erythrocyte membrane proteins. Interestingly, in a blood smear test, there were no significant alterations in erythrocyte morphology, but the patient displayed persistent unexplained normocytic anemia, which means that a lower number of red blood cells was observed. Although it seems likely that MPP1 has an important role during erythropoiesis, further studies will be necessary to explain the exact role of MPP1 in this process [97].

Complexes of a similar nature to MPP1-4.1R-GPC were also observed in non-erythroid cells, such as epithelial and neuronal cells. In epithelial cells, MPP1, MPP2 and MPP3 were shown to form a ternary complex with another member of the 4.1 family, 4.1B, and the transmembrane protein CADM1 (cell adhesion molecule 1, also known as IGSF4, NECL-2, SynCAM1, or TSLC1) [53,61,67,68,89]. CADM1, a member of one of the cell adhesion molecule families (CAMs), nectin-like molecules (Necl), is involved in the establishment of cell adhesion and is also known to play a role as a tumor suppressor in various human cancers [53,98]. Interestingly, CADM1 shares a strong homology of its cytoplasmic region with GPC, and pull-down assays indicated that its PDZ-binding motif is crucial for binding to MPPs [53]. In turn, the interaction of protein 4.1B with MPP1, MPP2 and MPP3 was shown to be independent of CADM1, and the binding site was mapped to a core region of the FERM domain of 4.1B [53]. Further studies showed that MPP1, MPP2 and MPP3 are located along the cell membrane in confluent HEK293 cultures, and their immunostaining pattern coincides with CADM1 and 4.1B [53]. However, only MPP2 co-localized with CADM1 and 4.1B at the cell–cell contact sites in a low-density culture of HEK293 cells and the MPP2-4.1B-CADM1 complex seemed to be linked to F-actin. The suppression of CADM1 expression in HEK293 led to mislocalization of both MPP2 and 4.1B, and negatively affected the epithelia-like structure formation, which was observed as a flat and enlarged morphology of cells [53]. HEK293 cells lacking CADM1 also presented thin actin filament (AF) bundles at the cell–cell contact sites. A similar negative impact on cell morphology of another epithelial cell line, Caco-2, was also observed under suppression of CADM1 expression. In contrast, the attempt to silence MPP2 or 4.1B expression did not cause any significant changes. This was explained as possible complementation by other proteins from MPP and 4.1 families. Interestingly, MPP2 mislocalization has been observed in two non-small-cell lung cancer (NSCLC) cell lines, NCI-H596 and SK-LU-1, characterized by a loss of CADM1 and 4.1B expression [53]. Moreover, these cell lines presented the transformed morphology, including disruption of cell–cell contact and disorganization of AF bundles. These results pointed to the possible role of the MPP2-4.1B-CADM1 complex in the formation of epithelial cell-like structure and might indicate its disruption as a possible cause of morphological transformation of cancer cells [53].

An example of a protein complex, similar to those previously described, was discovered in mouse sciatic nerves, where MPP6 was found in the complex with protein 4.1G, Lin-7 (a MAGUK protein, which is described below in more detail) and another member of the Necl family (CADM4) [84,99,100,101,102]. Immunostaining studies of MPP6 and CADM4 showed that, analogously to 4.1G, they can be found at the cell membrane of Schmidt-Lanterman incisures (SLIs) and paranodes [84,99]. In turn, the knockout of 4.1G caused the mislocalization of other proteins from SLI membranes [84,99]. Interestingly, western blot analysis showed a significant decrease compared to the control in total amount of MPP6 in 4.1G knockout nerves [84]. These results strongly resemble the situation observed for MPP1 and GPC in erythrocytes upon the absence of 4.1R [95]. Further electron microscopy studies revealed abnormalities in myelin internodes in 4.1G-deficient mice, represented as an increase in myelin thickness, comma-shaped myelin appearance and myelin membrane folding [101]. The absence of 4.1G protein also impacted the structure of paranodes, where the attachment of myelin tips was disrupted and axonal membrane swelling was observed. Defects in axonal membrane organization and myelin formation were also observed under the deletion of gene encoding CADM4 [103]. Moreover, mice deficient in 4.1G or CADM4 exhibited impairment of motor function and slower nerve-conduction velocity [101,103]. On the other hand, the absence of MPP6 did not affect the expression and localization of 4.1G or CADM4, thus showing that MPP6 is not essential for translocation of these proteins [102]. The analysis of the axon diameter in MPP6-deficient mice showed no changes in comparison to the control; however, the diameters of the nerve fibers (axon and myelin) were increased, thus implicating a possible role of MPP6 in the negative regulation of myelin formation [102]. On the other hand, the MPP6-deficient mice did not show any changes of motor function in comparison to the control. These results indicate that the MPP6-4.1G-CADM4 complex has a function similar to the ternary complex found in erythrocytes in the resistance against external mechanical forces and in maintaining SLI structure.

In mouse renal S1-S2 proximal tubules, MPP1 showed a similar staining pattern to protein 4.1B, indicating the same basolateral membrane localization of both proteins [54]. Further immunoprecipitation experiments indicated protein 4.1B as a protein partner of MPP1. The same studies showed that protein 4.1B binds to sodium bicarbonate cotransporter 1 (NBC1), a protein belonging to the family of SLC4 bicarbonate-absorbing transporters, which includes the erythrocyte membrane transporter AE1. Therefore, it was proposed that these proteins may form a complex at the basolateral surface of the kidney S1-S2 proximal tubules, although its existence needs further studies. Immunolocalization studies of MPP1 also demonstrated progressive changes in its distribution from the cytoplasm to the basolateral membrane, which correlated with the maturation of the renal proximal tubules, thus implying a possible role of MPP1 in the maturation of the proximal tubule [54]. As other studies report that NBC1 displays similar progressive maturation, Terada et al. also hypothesized that maturation of these proteins might reflect the development of acidifying mechanisms in the proximal tubule [54].

Proteins belonging to the ezrin-radixin-moesin (ERM) family, another representative group of the FERM domain-containing proteins, were also found to associate with MPPs. In epithelial cells, one of the members of the ERM family, ezrin, was shown to form a complex with MPP5 [83,104,105]. Ezrin is a major component of microvilli, where it links actin microfilaments with plasma membrane [105]. Detailed studies on gastric parietal cells showed interaction of the N-terminal part of ezrin with MPP5, as indicated by the pull-down experiments and their co-localization in the apical vacuole membrane of the resting and secreting parietal cells [83]. The interacting regions correspond to the FERM domain of ezrin and core domains of MPP5, which include the D5 domain. It may indicate that this mechanism of interaction is analogous to MPP1-4.1R. The MPP5 knockdown studies demonstrated the loss of apical localization of ezrin, the latter being diffused through the cytoplasm with no co-localization with F-actin. Moreover, after stimulation with histamine, the vacuole diameter in knockdown cells resembled that of resting parietal cells [83,106]. These results point to the crucial role of the interaction between ezrin and MPP5 in the remodeling of the apical membrane connected with parietal cell secretion.

Another member of the ERM family, found to interact with MPPs, is merlin (moesin-ezrin-radixin-like protein), also called neurofibromin 2 or schwannomin [58,107]. Merlin is a protein encoded by the *NF2* gene, the inactivation of which was connected with the development of tumors in humans and mice [107]. To date, various mechanisms underlying the tumor-suppressor activity of merlin have been identified, including increased apoptosis and negative regulation of cell growth or proliferation [107]. Despite predominant expression in Schwann cells, merlin was also identified in erythrocyte membranes, and the way of its membrane association seemed to be similar to lipid anchored proteins, such as MPP1 [108]. These results and the fact that merlin contains in its structure two possible binding sites for MPPs, the FERM domain and the C-terminal PDZ domain-binding sequence, have led to a plausible interaction between merlin and MPP1 being investigated [58]. Pull-down assays and surface plasmon resonance (SPR) analysis, performed on bacterially expressed recombinant proteins, showed that MPP1 specifically interacts with the N-terminal part (containing the FERM domain) of merlin with a K_D_ value of ~3.7 nM [58]. Further immunoprecipitation studies on human erythrocyte ghosts demonstrated that merlin co-precipitated with MPP1, demonstrating the existence of their mutual interaction in erythrocyte membranes. Moreover, immunohistochemistry of MPP1 and merlin in the rat sciatic nerves showed the co-localization of both proteins in non-myelin-forming Schwann cells, which might suggest the presence of this complex in non-erythroid cells as well. Although the functional role of this complex awaits discovery, the direct interaction of MPP1 with the purified recombinant FERM domain of merlin may indicate binding of MPP1 to the unphosphorylated form of merlin [58]. In this form, merlin was demonstrated to be in the closed conformation, which was found to serve as a tumor growth suppressor. Therefore, Seo et al. suggested that the MPP1-merlin complex may take part in cell polarity mechanisms and through cytoskeletal reorganization might regulate the merlin tumor-suppressor activity during processes such as cell division or proliferation [58]. However, as it was noted by the authors, this hypothesis needs further investigation.

As presented above, MPP-FERM complexes occur commonly in erythroid as well as in non-erythroid cells and through interaction with transmembrane proteins, they anchor the actin cytoskeleton to the cell membrane. These ternary linkages are essential for membrane remodeling, and thus in establishing a proper cell structure/shape. Any disruption of interactions between proteins involved in the complex led to significant structural and physiological consequences, among others, elliptic shape of erythrocytes or overmyelination of nerves. To date, the most extensively studied complex formed by MPPs is the ternary complex found in erythrocyte membranes formed by MPP1 with 4.1R and GPC. Other MPPs were shown to form complexes in a similar pattern and their D5 domain was demonstrated to interact with the FERM domain of FERM family members. Interestingly, MPP3 was shown to interact with 4.1B, although it lacks characteristic features of the D5 domain in its structure (Figure 1a), which suggests the existence of alternative mechanisms of interaction in comparison to other MPPs.

## 3. MPPs in Complexes with Other MAGUK Members: Lin-7, Discs Large Homolog 1 (Dlg1) and Postsynaptic Density Protein 95 (PSD-95)

Among the characteristic features of the MAGUK superfamily are interactions between other members of the group, and MPPs are not an exception. MPPs were shown to form complexes with discs large homolog 1 (Dlg-1), postsynaptic density protein 95 (PSD-95) and Lin-7 [5,37,60,73,90]. Dlg-1 is a multidomain protein that consists of an L27 domain, three PDZ domains, an SH3 domain and a GUK domain and is an essential scaffolding protein involved in the process of adherens junction assembly [109]. PSD-95, as suggested by its name, mostly localizes at the postsynaptic densities where it plays a crucial role in mediating intracellular signaling and maturation of the synapses [110]. Analogous to Dlg-1, PSD-95 contains three PDZ domains, an SH3 domain and a GUK domain [111], while its alternatively spliced isoform, PSD-95β, contains an additional L27 domain at the N-terminus [111]. Lin-7, also known as Mals (mammalian Lin-7) or Veli (vertebrate Lin-7), is expressed as three isoforms: Lin-7A, Lin-7B and Lin-7C [63,112]. All of them are predominantly found in brain neurons, while Lin-7C is also commonly expressed in epithelial cells [112]. Lin-7 is a small protein and contains only two domains, L27 and PDZ [113].

Both MPP5 and MPP6 (PALS1 (protein associated with Lin-7) and PALS2, respectively) were found to co-localize with Lin-7 at the membrane of MDCK cells [5]. Knockdown of Lin-7 in MDCK cells showed the importance of this protein for regulation of MPP5 expression. Under conditions of Lin-7 deficiency, MPP5 was unstable and had a higher rate of degradation, which resulted in structural defects in the tight junction and their delayed formation. That could be rescued with overexpression of the Lin-7 L27 domain and, interestingly, also with overexpression of MPP5 [113]. The above results imply that Lin-7 does not participate in tight junction formation, but stabilizes MPP5 during the process, as MPP5 CC (coiled coil) and L27N domains were shown to be essential for targeting the tight junctions [5,31,113]. Analyses of the MPP5 L27C domain and Lin-7 L27 domain interaction indicate that this process may be mediated by hydrogen bonds between the charged residues and hydrophobic interactions in the core of the complex [5,31,114].

Tseng et al. observed that MPP6 might be involved in the assembly of Lin-7 protein complexes in epithelial and neuronal cells. Indeed, further studies confirmed that MPP6 and Lin-7 form, as already mentioned, a molecular complex with CADM4 and 4.1G in mouse peripheral neurons [84,99,100,101,102]. In MPP6-deficient mice, Lin-7 expression was downregulated and absent in SLIs in Schwann cells, thus indicating MPP6 as a regulator of Lin-7 expression and localization. Moreover, the myelin sheath of the sciatic nerves was thicker, which suggested the possible role of the MPP6-Lin-7 complex in myelin formation [102]. Similar dependence was seen in spermatogonia and in early spermatocytes of mice seminiferous tubules, where immunolocalization and western blot analysis in MPP6-deficient mouse testis showed a lower intensity signal from Lin-7 [86]. Those observations confirmed the new role of MPP6 as a negative regulator of myelination and pointed out the role it plays in Lin-7 transport [86,102].

In the mammalian retina, two MPPs were detected in a complex with Lin-7: MPP5 at the outer limiting membrane (OLM) of the photoreceptor synapse and MPP4 at the synaptic terminals of the outer plexiform layer (OPL), which is on the opposite side of the intercellular contact. The interaction of MPP4-Lin-7C was then confirmed with co-immunoprecipitation from retina lysates and pull-down assay, where all isoforms bound to MPP4 [73,74]. The binding between the Lin-7 L27 domain and the homodimer of MPP4 L27 domains was most efficient, but L27C was mainly responsible for the interaction [74]. The same mechanism applies to complexes formed by Lin-7 with MPP3 in MDCK cells and with MPP7 in epithelial cells [7,62].

Although MPP7 associates in vitro with all Lin-7 isoforms, only Lin-7A or Lin-7C was found in a complex with MPP7, as was another MAGUK, Dlg-1, in HEK 293 cells [90]. It is worth emphasizing that Dlg-1 co-precipitated with MPP7 and Lin-7 only when all three proteins were co-expressed. Moreover, it was demonstrated that MPP7 and Lin-7 increase each other’s expression and stability, which seems to be similar to the relationship of Lin-7 with MPP5. Binding through MPP7 L27N (Figure 1a) was crucial for localization of Dlg-1 in the tight junctions, which was enhanced by interaction with the Lin-7-MPP7 dimer [90]. Depletion of either MPP7 or Dlg-1 had a negative impact on the assembly of functional cell junctions, pointing to the functional role of the complex in the formation of these structures [13]. Although the MPP7 L27N domain may be sufficient in assuring Dlg-1 plasma membrane localization, both L27 domains of MPP7 are necessary for the interaction [37,90]. Similar results concerning the interaction mechanism with Dlg-1 were obtained for MPP3 in MDCK cells [90].

Although initially MPP3 and MPP4 were found in a ternary complex with Dlg-1 in HEK 293 cells, more detailed research proved that they form separate complexes with different isoforms of Dlg-1 at the OPL of photoreceptor synapses [8]. Those differences may be related to a linking protein that may exist only in HEK 293 cells or to the recruitment of MPPs to distant subdomains of the retina. This finding did not rule out the existence of Dlg1-MPP3 or Dlg-1-MPP4 complexes, but merely pointed to their various functional roles at photoreceptor synapses [8].

A study by Aartsen et al. stressed the crucial role of MPP4 in maintaining PSD-95 at the photoreceptor presynaptic membranes. In mice expressing only two L27 domains of MPP4, protein levels of PSD-95 were strongly reduced at the photoreceptor synapses, while in MPP4 null mice, changes in electroretinograms and complete loss of PSD95 from the photoreceptor terminal were observed [73,75]. Further research led to the identification of two PSD-95 isoforms (PSD-95α and PSD-95β) and additional proteins that co-localize with the MPP4-PSD95 complex, such as plasma membrane Ca^2+^ ATPases (PMCAs) [71,73]. The interaction between MPP4 and PSD-95β was linked to the heterodimerization of their L27 domains, and this complex was proved to be involved in the two-step process of proper localization of the PMCAs [71,72,73]. First, the homodimer of MPP4 L27 domains recruits PMCAs toward the photoreceptor terminal. Then, the C-terminal part of MPP4, along with PSD-95β, stabilizes its localization at the presynaptic plasma membrane [68,71]. Together with PSD-95β, MPP4 may be assumed to be an essential part of the complex responsible for proper functioning of signal transducers (e.g., PMCA) at the photoreceptor synapse.

In rats, the synaptic plasma membrane and postsynaptic density (PSD) fractions of the forebrain were shown to be enriched in MPP1 [60]. Its high expression was even detected at the early postnatal stage. Immunostaining of cultured cortical neurons demonstrated partial co-localization of MPP1 with PSD-95 and to a lesser extent with calcium/calmodulin-dependent serine protein kinase (CASK), a presynaptic membrane protein, thus suggesting postsynaptic localization of MPP1 [60]. Further studies showed that MPP1 interacts with many PSD proteins, among others, PSD-95, CASK, guanylate kinase-associated protein (GKAP) and Dlg-1 [60]. Detailed studies of MPP1-PSD-95 interaction indicated D5 and GUK domains of MPP1 as a binding site for PDZ domains of PSD-95 [60]. Therefore, it was proposed that MPP1 might be one of the postsynaptic membrane scaffold proteins involved in early stages of synaptogenesis, linking various PSD molecules needed for synaptic adhesion and postsynaptic signaling [60].

Similar results were obtained for MPP2 in neuronal synapses [60,61]. The immunostaining signal for this protein matched with the signal of PSD-95, proving postsynaptic localization of MPP2. Further co-immunoprecipitation and pull-down experiments confirmed the binding of PSD-95 with MPP2 through the SH3 and GUK domains of the latter [61]. These results suggested an interaction mechanism similar to that found for MPP1, as the D5 domain is located between SH3 and GUK domains [60]. The same study also indicated a second major component of the PSD, GKAP, as a protein partner of MPP2 that exhibited the same interaction mechanism as MPP2-PSD-95 [61]. Further studies on possible function of the MPP2-PSD-95-GKAP complex led to the identification of another MPP2 protein partner, CADM1 (Figure 2b) [61]. Direct binding of the C-terminal part of CADM1 by MPP2 suggests that the latter may bridge other proteins with components of the PSD, such as PSD-95 and GKAP in neurons (Figure 2b) [61]. In the case of MPP1, it was proposed that it is one of the postsynaptic scaffold proteins in the early stage of synaptogenesis, linking various PSD molecules needed for synaptic adhesion and postsynaptic signaling [60].

Interactions between MPPs and other MAGUKs can be found in nearly every cell type, as they have been shown to be involved in the establishment of cell junctions, retinal communication, cell polarity and cell signaling. Loss of any of the complexes mentioned above may have negative implications for the proper functioning of the cell. In most cases, MPP-MAGUK complexes present a similar mechanism of interaction that is based on dimerization of L27 domains. In the case of MPP5-Lin-7 and MPP7-Lin-7, some resemblance in mutual influence on each other’s stability and/or expression can be noted.

## 4. MPPs in Complexes with Conserved Cell Polarity Complexes (Crumbs Complex and Partitioning Defective (Par) Complex)

The establishment and maintenance of cell polarity are crucial for eukaryotic cells as they are connected to a large number of biological functions and events [115,116]. For epithelial and neuronal cells, maintaining apicobasal polarity is extremely important for proper functioning [117]. This is accomplished with the help of scaffolding proteins, such as MPPs, which fulfill a critical role in organizing and maintaining protein functional complexes located at the cell membranes. Some of them are evolutionarily conserved complexes such as the Crumbs complex or the partitioning defective (Par) complex. The former consists of Crumbs (CRB), MPP5 and Pals1-associated tight junction protein (PATJ) and the latter consists of Par3, Par6 and atypical protein kinase C (aPKC) (Figure 2c) [118,119].

Within the Crumbs complex, MPP5 mediates the interaction between two other members: PATJ protein (Figure 1E) and CRB [31,45,79]. The later one is an evolutionary conserved apical transmembrane protein that was first discovered in *Drosophila* and was shown to be expressed as three isoforms in vertebrates. CRB1 is mainly expressed in the retina; CRB2 is expressed in the uterus, testis, eye, brain and embryonic tissue; and CRB3 is the most common isoform in epithelial cells [120,121].

The C-terminal ERLI motif of CRB was indicated as a binding site of the MPP5 PDZ domain (Figure 1D) [80,120,122]. However, using isothermal titration calorimetry (ITC), it was demonstrated that apart from the PDZ domain, MPP5 SH3/GUK domains are needed to form a platform for high affinity (K_D_ ∼70 nM) interaction with CRB3 [25]. Knockdown of MPP5 in MDCKII cells led to decreased interaction between CRB and Par6, aPKC was not properly targeted to the tight junctions and the delivery of E-cadherin to the cell surface was altered. As a result, delay in tight junction formation, defects in polarity and inability to form a luminal cyst in cells occurred [31,34,79,120,123]. Moreover, it was reported that small envelope protein (E) from SARS coronavirus binds to the PDZ domain of MPP5. Findings from MDCKII cells overexpressing protein E showed a delay in tight junction formation just like in the cells with knocked-down *MPP5*. This, in turn, suggests the possibility that the damage observed in lung biopsies from infected patients may be the result of suppression of MPP5-CRB interaction by SARS-E [124].

Participation in the Crumbs complex was also demonstrated for MPP7 in epithelial cells and for MPP1, MPP3 and MPP4 in the retina [8,35,37,125]. Two mechanisms have been shown to be necessary for the proper association of MPP7 with the plasma membrane: interaction with Dlg-1 via L27 domains, which was described earlier, and interaction dependent on the CRB3 ERLI motif and the MPP7 SH3-D5 domains, which were indicated as MPP5-binding sites [37,62]. Overexpression of all three proteins in HEK 293T cells revealed that MPP7 interacts with CRB3 to a much lesser extent than MPP5. Thus, these results suggest that MPP5 draws MPP7 toward the membrane, thereby enabling its interaction with CRB3 [37].

There are some parallels between interactions of MPP3 and MPP4 with the Crumbs complex. Both of them are recruited to the complex by direct interaction with MPP5 and form complexes at the subapical region (SAR) adjacent to adherens junctions at the OLM in the retina. The binding sites of MPP3 and MPP4 were found to be GUK and SH3-GUK domains, respectively [8,35]. However, results obtained from immunostaining showed a much stronger signal from MPP4 alone than from MPP3, at the OPL. Surprisingly, in co-immunoprecipitation experiments from HEK293 cells, MPP3-CRB1 binding was only detected in cells overexpressing all three proteins. These differences in main localization are connected to the proposed roles of these protein complexes. The complex with MPP4 is thought to be involved in the pathway which establishes the polarity of photoreceptors, whereas MPP3 is more likely to maintain and organize an intracellular scaffold in the retina [8,35].

Similar to complexes described above, MPP5-MPP1 and MPP1-whirlin were also found co-localizing at the OLM of the photoreceptors [26]. Whirlin is a multi-PDZ protein and a part of an important scaffolding protein complex related to the Usher syndrome, which is manifested by deafness accompanied by progressive loss of vision [126,127]. As this disorder is connected to the retina, where all three proteins are present, it may indicate that the Crumbs protein complex is linked with the Usher protein network via interaction between MPP5 and MPP1 [26]. The MPP5-MPP1 SH3-D5 and SH3-GUK domains were suggested as binding sites. In the case of MPP1 and whirlin, the binding sites were GUK and PDZ domains, respectively [26].

Key processes regarding the regulation of polarized cell proliferation, such as, stabilizing balance between cell proliferation and apoptosis or controlling the cell number or organ size, are regulated by the Hippo pathway [128,129,130]. Changes such as excessive cell proliferation or inhibition of apoptosis may be associated with tumor formation [128,130,131]. The interconnection between the Crumbs complex and the Hippo pathway, one of the evolutionarily conserved signaling pathways, was first discovered in *Drosophila* [132,133]. In mammalian cells, association of the Crumbs complex was shown to activate large tumor suppressor (Lats) kinase, which leads to phosphorylation of yes-associated protein 1 (YAP1) and cytoplasmic seclusion of YAP1 and tafazzin (TAZ) by recruiting them to the apical membrane [132]. Knockdown of MPP5 leads to deregulation of the Hippo pathway by decreasing YAP1 phosphorylation and increasing its nuclear localization, which causes hyperactivation of downstream target genes [82]. Mice with *MPP5* knockout developed proteinuria and died within 4-6 weeks after birth [134]. Moreover, another crucial signaling pathway, TGF-β signaling, was affected by this knockout. Like Hippo signaling, TGF-β signaling is important in cell proliferation and apoptosis. Target genes of both of those pathways were significantly upregulated in MPP5 knockout mice. This connects to the results obtained from the crosstalk experiments in epithelial cell lines, showing that MPP5 works as an upstream regulator between these two signaling pathways [134].

There is another protein from the MPP family which plays a role in Hippo signaling. MPP7 with its protein partner angiomotin (AMOT) was shown to form a ternary complex with YAP1 protein, when co-expressed together in HEK293T cells. But unlike MPP5, MPP7-YAP1 interaction is important for YAP1 nuclear localization in activated muscle stem cells [92]. In 2019, New et al. reported MPP7 as a novel component of the regulatory mechanism of autophagy and cell survival in pancreatic ductal adenocarcinoma (PDAC). They stated that activation of YAP1 by MPP7 is essential for the process of YAP1-mediated autophagy [42].

MPP5 connects the Crumbs complex with another polarity complex, the Par complex. Both were found to be associated via MPP5 CC-L27N domains, the Par6 PDZ domain and the Cdc42/Rac-interactive binding (CRIB)-like motif [77]. Interestingly, the L27N domain is also the binding site for PATJ, but these two proteins do not interact directly with each other. Moreover, increase of MPP5-Par6 and decrease of MPP5-PATJ binding were seen in pull-down experiments from HEK293 cells co-transfected with increasing amounts of Par6 with stable amounts of PATJ. Changes in the MPP5 conformational state or steric hindrance were suggested as an explanation of this phenomenon [78]. On top of that, a small GTPase, Cdc42, which binds Par6, acts as a regulator and influences protein binding. MPP5 and Par6 expressed in Cos7 cells showed a stronger association when co-expressed with the constitutively active Cdc42 (Cdc42-GTP) [77].

As presented above, with the exception of MPP2 and MPP6, most of the MPP family members are involved in regulation of cell polarity. Through SH3-D5 or SH3-GUK domains, MPP5 recruits other MPPs to the cell junctions and bridges their interaction with CRB in different cell types, thus allowing establishment and maintenance of apicobasal polarity. MPP5 also plays a role in crosstalk between Hippo-mediated and TGF-β-mediated signaling as a dose-dependent upstream regulator. The other MPP which has a regulatory role in connection to Hippo-mediated signaling is MPP7. Although they have opposing effects on the signaling cascade, MPP5 together with CRB precludes the nuclear localization of YAP1, while MPP7 activates YAP1 and leads to its accumulation in the nucleus.

## 5. MPPs in Complexes with Flotillins

Recently, a member of the MPP family, MPP1, was shown to have another role in erythrocyte and erythroid cell membranes, besides its function as a scaffolding protein in the aforementioned ternary complex [10,38,57,135,136]. Studies on erythroid cells suggested that MPP1 may have a wider role in plasma membrane organization, in particular, in membrane raft formation [136]. Using the erythrocyte progenitor cell line, HEL (human erythroleukemia), it turned out that the lack of palmitoylated MPP1 in the plasma membranes was the cause of these phenomena [10]. The inhibition of MPP1 palmitoylation or silencing of MPP1 gene expression in HEL cells led to a dramatic decrease of isolated detergent-resistant membranes (DRMs), which was correlated with significant reduction of membrane ordering parameters, such as membrane fluidity [10]. DRMs are insoluble in non-ionic detergent membrane fragments isolated from cells, which are thought to resemble, in part, membrane rafts in their protein and lipid composition. Found in the intact cells, membrane rafts are nanoscopic membrane domains enriched in cholesterol, sphingolipids and proteins with a glycophosphatidylinositol (GPI) anchor or linked to the membrane with acyl chains, which are formed from unstable and highly dynamic ordered nano-assemblies [137]. Although the terms ‘DRMs’ and ‘rafts’ cannot be considered as interchangeable, as they are not the same structure, DRM isolation is still a useful tool to study lateral membrane organization [137,138]. In this study, knockdown cells and cells with inhibited palmitoylation showed lower levels of cholesterol and MPP1 in isolated DRMs, in comparison to control cells [10]. These results point to the pivotal role of MPP1 palmitoylation for its localization in membrane rafts. Further studies indicated that changes observed in membrane organization affected the function of membrane rafts, which led to a reduction in MAP kinase signaling pathway activation via raft-dependent RTK receptors such as the insulin receptor (IR) or c-kit receptors [10,135]. It was shown that inhibition of signal transduction occurs at the level of H-Ras, a small GTPase, whose function depends on its localization within raft domains, indicating a novel functional link between H-Ras activation and MPP1-dependent membrane raft formation [135]. Altogether, these results uncover a novel role of palmitoylated MPP1 in regulation of raft domain formation.

**Figure 3:**
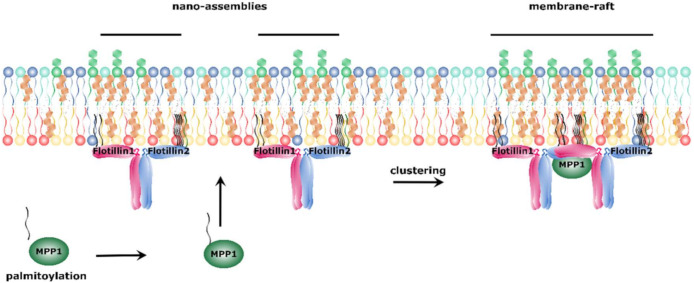
Proposed mechanism of functional membrane raft formation. Palmitoylated MPP1 binds to the flotillins present in cholesterol and sphingomyelin-enriched nano-assemblies. This binding initiates oligomerization of flotillins and coalescence of nano-assemblies into membrane rafts.

In an attempt to answer the question how palmitoylated proteins may affect membrane organization, novel MPP1 protein partners—flotillin 1 and flotillin 2—were identified in erythrocytes [57]. Flotillins, also known as reggie proteins, are highly conserved and ubiquitously expressed proteins that belong to the group of stomatin, prohibitin, flotillin, HflC/K (SPFH) domain-containing proteins [139,140,141] and are known as membrane raft protein markers [142]. In contrast to multidomain MPPs, their structure can be described with the N-terminal SPFH domain that mediates interaction with cholesterol-rich membrane fragments by post-translational modifications (palmitoylation and myristoylation) and the C-terminal flotillin domain that is characteristic for flotillins and contains coiled-coil regions responsible for flotillin oligomerization [141,143]. Oligomerization of flotillins is thought to play an essential role in the assembly and organization of cholesterol and sphingomyelin-enriched nano-assemblies, and their further transformation toward membrane rafts. Most recently, we were able to provide kinetic parameters of MPP1-flotillin interaction and identify the “flotillin-binding site” within the MPP1 D5 domain (Biernatowska and Olszewska et al., to be published). Remarkably, flotillins do not possess any PDZ-binding motif and the interaction with MPP1 is independent of protein 4.1R [57]. As flotillins are markers of membrane rafts, a question emerged on how MPP1 is connected to lateral membrane organization. One of the concepts regarding their formation involves interactions between raft-associated proteins [136,144,145]. This may be the mechanism explaining the role of MPP1-flotillin interaction in this process. Based on the aforementioned results, our group proposed that palmitoylation of MPP1 triggers the clustering of pre-existing flotillin-associated nano-assemblies into functional membrane rafts (Figure 3). The question how MPP1 palmitoylation impacts the stabilization of those assemblies remains open.

The discovery of flotillins as binding partners of MPPs sheds new light on the role which this family plays in molecular organization of cell membranes. Previously described MPP-mediated complexes demonstrate a similar formation pattern, and they have a similar function in different cells. It is possible that, depending on the cell type, other MPPs or MAGUKs, through interaction with flotillins, play a role similar to MPP1 in the organization of lipid nano-assemblies into membrane rafts. In turn, palmitoylation was indicated to be crucial not only for MPP1 raft partitioning, but also for its possible role in lateral membrane organization. Previously, palmitoylation was shown to regulate the function of other MAGUKs [144,146,147,148]. Thus, it is likely that this modification of MPPs is indispensable for the proper function of MPP family members.

## 6. Summary

MPPs are scaffolding proteins from the MAGUK family, which can be found at the center of many biological processes—from the role in establishing cell structure, through maintaining cell junctions, cell polarity and regulation of cell signaling, to the probable main role in membrane raft formation mechanism. As ubiquitously expressed proteins with a multidomain structure, they have nearly unlimited possibilities for interactions.

In most cases, they can be found in ternary complexes near the plasma membrane or in structures connected to it. Complexes with members of the 4.1 superfamily serve as a link between cell membrane and cytoskeleton, thus playing a crucial role in the establishment of cell structure. Loss of interaction between proteins found in the complex is a frequent trigger of incorrect localization of MPPs in the cell and has significant physiological implications. The best-known example is a tripartite complex found in erythrocyte membranes formed by MPP1, 4.1R and GPC. Other MPPs take part in similar complexes, and the D5 domain was indicated as a binding site of FERM proteins. Another crucial complex formed by MPPs is with the proteins belonging to the CAM family. Their interactions are similar to those with GPC and are based on the PDZ-binding motif recognizing PDZ domains of MPPs. Those complexes are mostly found at cell junctions, and their disturbance causes loss of epithelial cell-like structure and possibly is connected with morphological transformation of cancers. In a similar manner to complexes with protein 4.1, complexes with MPP5 also serve as a molecular bridge. MPP5-based complexes connect other proteins, including MPPs, to the complexes located at the cell junctions, mostly to the CRB protein. Those interactions also demonstrated that binding between MPPs is mediated by their SH3-GUK domains. Thus, MPPs have the ability to bind other members of the MAGUK family. Such interactions are represented by complexes with Dlg-1, Lin-7 and PSD-95. The mechanism behind them is based on the homodimerization of L27 domains. Complexes formed by MPPs with other MAGUKs were observed to have a main function in the maintenance of cell junctions and apicobasal cell polarity.

Last but not least, an emerging role of MPP1 in lateral membrane organization seems to be of particular interest. The exact mechanism of membrane raft formation is still a matter of debate. However, interactions between MPP1 and raft marker proteins, flotillins, represent a novel, interesting concept of how organization of such domains might be controlled in living cells. As MPPs and flotillins are ubiquitously expressed and involved in similar cellular processes, mutual MPP-flotillin interactions might be an important factor in regulating the lateral organization of the cell membrane, where MPPs can act as molecular switches inducing oligomerization of raft-associated proteins. Moreover, as shown for MPP1, the palmitoylation process and its impact on protein function seem to be interesting points for further exploration as they are the major characteristic features of all MPP members.

Despite the significant progress toward understanding the involvement of the MPP family in various cellular processes that was made in recent years, there are still a lot of unanswered questions concerning the exact function of MPPs in some biological processes. Therefore, further research is required to fully understand their multifaceted nature.

## Figures and Tables

**Table 1:** List of MPP protein partners.

MPP Protein	MPP Binding Domain (If Identified)	Protein Partner	Domain/Motif of Partner Protein Binding MPP (If Identified)	Method Used for Identification	Source
**MPP1**	PDZ (21 aa at N-terminus)	Glycophorin C (GPC)	12 aa on C-terminal, type II PDZ-binding motif (EYFI)	SPR, overlay assay, ELISA, resonant mirror detection	[47,48,49,50]
	D5/HOOK	4.1R	FERM domain	SPR, overlay assay, ELISA, resonant mirror detection	[47,48,50,51,52]
		4.1B	FERM domain	Pull-down, co-IP	[53,54]
	GUK + C-terminal atypical PDZ-binding motif	Whirlin	PDZ3 and proline-rich domain	Yeast two-hybrid system, pull-down, co-IP	[26,55]
	GUK	Gelsolin	Gelsolin-like repeats	Pull-down, co-IP	[56]
	SH3-GUK	MPP5	SH3-D5	Yeast two-hybrid system, pull-down, co-IP	[26]
	D5	Flotillin 1		Co-IP, pull-down, overlay assay, SPR, ELISA, PLA	[57], Olszewska et al. to be published
	D5	Flotillin 2		Co-IP, pull-down, overlay assay, SPR, ELISA, PLA	[57], Olszewska et al. to be published
		Merlin	FERM domain	SPR, co-IP, pull-down	[58]
	PDZ	ABCC4	C-terminal PDZ-binding motif (ETAL)	Co-IP, PLA	[59]
	D5-GUK	PSD-95	PDZ	Pull-down, co-IP	[60]
	D5-GUK	Dlg-1/SAP97		Pull-down, co-IP	[60]
	D5-GUK	CASK		Pull-down, co-IP	[60]
	D5	actin		Pull-down, co-IP	[60]
	D5	CaMKII		Pull-down, co-IP	[60]
**MPP2**	SH3-D5-GUK	PSD-95		Yeast two-hybrid system, pull-down, Co-IP	[61]
	SH3-D5-GUK	GKAP		Yeast two-hybrid system, pull-down, co-IP	[61]
	PDZ	CADM1/Necl-2/SynCAM1	C-terminal type II PDZ-binding motif (EYFI)	Yeast two-hybrid system, pull-down, co-IP, ITC	[53,61]
		MPP2		Co-IP	[61]
		4.1B	FERM domain	Pull-down, co-IP	[53]
		Dlg-1	L27 domain	Co-IP	[7,62]
	L27C	Lin-7A		Pull-down, co-IP + mass spectrometry	[7,63]
		Lin-7C		Co-IP + mass spectrometry	[7]
	SH3-D5-GUK	SK2-L	N-terminal domain	Pull-down, co-IP + mass spectrometry	[7]
	PDZ	c-Src	PDZ-binding motif (GENL)	Co-IP	[64]
		Caspr2	Type II PDZ-binding motif EWLI and juxtamembrane protein 4.1-binding motif	Pull-down, co-IP	[65,66]
**MPP3**	PDZ	CADM1/Necl-2	Type II PDZ-binding motif (EYFI)	Yeast two-hybrid system, pull-down, co-IP	[53,67,68]
		4.1B	FERM domain	Pull-down, co-IP	[53]
		CADM3/Necl-1	Type II PDZ-binding motif (EYFI)	Yeast two-hybrid system, co-IP	[69]
	PDZ	Nectin-1	Entire C-terminal part, containing type II PDZ-binding motif (EWYV)	Yeast two-hybrid system, co-IP	[70]
	PDZ	Nectin-3	Type II PDZ-binding motif (EWYV)	Yeast two-hybrid system	[70]
	GUK	MPP5	SH3-D5	Co-IP	[8]
	Both L27	Dlg1	L27 domain	Pull-down, co-IP, far WB	[8,62,68]
	L27C	Lin-7		Co-IP	[62]
	PDZ	(5-HT)2C	C-terminus RISSV; EKVCV	Co-IP, pull-down	[36]
**MPP4**	L27	PSD-95β		Co-IP	[71,72]
		Dlg-1/SAP97		Co-IP	[73]
	L27C-L27N	Lin-7	L27	Co-IP	[74]
		CRB (CRB1)		IP	[35]
	L27C-L27N	PMCA		Yeast two-hybrid; IEM (mouse/mutant mouse)	[71,75]
	C-terminal	PMCA		Yeast two-hybrid; IEM (mouse/mutant mouse)	[71,75]
		MUPP1		IP	[76]
	GUK	MPP5	SH3-D5	Yeast two-hybrid	[35]
**MPP5**	CC/L27N	Par6	PDZ	IP; pull down	[77,78]
	L27N	PATJ/INADL	L27	IP	[31]
	L27N	MUPP1/MPD2/	L27	IP	[31]
	L27C	Lin-7		IP	[5]
	PDZ-SH3-GUK	CRB	ERLI motif (COOH domain)	IHC; IP; ITC	[25,79,80]
	PDZ-SH3	GABA transporter (GAT1)	Multiple sites of contact: (1) AYI motif (COOH domain; “type II PDZ-binding motif”); (2) Between 549–576 aa	Yeast two-hybrid, co-IP, pull-down	[81]
	SH3-D5	MPP1	SH3-GUK	Yeast two-hybrid	[26]
	SH3-D5	MPP3	GUK	Co-IP	[8]
	SH3-D5	MPP4	GUK	Yeast two-hybrid	[35]
	PDZ-SH3-GUK	MPP5	PDZ-SH3-GUK	Crystallography	[25]
		MPP7	SH3-D5	IP	[37]
		Taz	WW-PDZ	IP	[82]
	C-terminal (181–675 aa; PDZ-SH3-D5-GUK)	Ezrin	N-terminal (1–50) (FERM domain)	Affinity precipitation of PALS1 and ezrin; pull-down	[83]
**MPP6**		4.1 G		Co-IP	[84]
		mLin-2/CASK		Co-IP	[85]
	L27C	Lin-7	L27	Co-IP, pull-down	[5,86,87]
		c-Src		Co-IP	[88]
		CADM3/Necl-1	Type II PDZ-binding motif EYFI	Yeast two-hybrid system, co-IP	[69]
	PDZ	CADM1/Necl-2	Type II PDZ-binding motif EYFI	Yeast two-hybrid system, co-IP, pull-down (affinity chromatography)	[69,89]
		Caspr2	Type II PDZ-binding motif EWLI and juxtamembrane protein 4.1-binding motif	Pull-down	[65]
**MPP7**	L27N	Dlg-1/SAP97	L27 domain	Co-IP	[37,90]
	L27C	Lin-7	Single L27	Co-IP	[37,90]
	SH3-D5	MPP5		Co-IP	[37,91]
		Caspr2	Type II PDZ-binding motif EWLI	Pull-down	[65]
		PATJ		PLA	[92]
		AMOT		PLA	[92]

Abbreviations: SPR, surface plasmon resonance; ELISA, enzyme-linked immunosorbent assay; co-IP, coimmunoprecipitation; PLA, proximity ligation assay; far WB, far-western blot; IEM, immune electron microscopy; ITC, isothermal titration calorimetry; IP, immunoprecipitation; IHC, immunohistochemistry.
